# Outcomes of Surgical Excision and High-Dose-Rate Brachytherapy for Earlobe Keloids

**DOI:** 10.29252/wjps.10.1.78

**Published:** 2021-01

**Authors:** Pedro Fuenmayor, Hector Quiñonez, Reinaldo Salas, Zoe Pujadas

**Affiliations:** 1 Plastic and Reconstructive Surgery Department, Hospital Universitario de Caracas, Universidad Central de Venezuela, Los Chaguaramos, Distrito Capital, Venezuela.

**Keywords:** Earlobe keloid, Excision, High-dose-rate brachytherapy, Plastic surgery

## Abstract

**BACKGROUND:**

Radiotherapy as an adjuvant therapy to surgical resection has shown variable rates of recurrence treating earlobe keloids. The purpose of this study was to describe our experience with surgical excision followed by high-dose-rate brachytherapy and present our outcomes after 24 months of follow-up.

**METHODS:**

Retrospective chart of 14 patients with 14 earlobe keloids treated with surgical excision followed by high-dose-rate brachytherapy, between January 2015 and May 2016 were enrolled. Database included demographics, Fitzpatrick skin type, laterality, lesion size, and follow-up visits information. Outcomes were assessed in terms of keloid recurrence rates, complications, and patient subjective aesthetical result satisfaction after 24 months of follow-up.

**RESULTS:**

All procedures were completed without complications. Three patients experienced keloid recurrence after 6 (14.28%) and 12 months (7.14%). Three patients experienced mild signs of self-limited post-radiation dermatitis. Self-assessment of aesthetical result was considered “very good” in 71.43% of patients.

**CONCLUSION:**

Surgical excision followed by high-dose-rate brachytherapy is secure and effective to treat earlobe keloids, and can be considered a first line combined treatment. Larger clinical trials comparing different irradiation protocols are still needed.

## INTRODUCTION

Keloid and hypertrophic scars are fibrous growths characterized by excessive collagen deposition located in a previous skin injury site. Keloids are complex conditions in which different ethnic groups have variable susceptibilities to develop the disease. Prevalence between populations might reflect different genetic risk factors.^1^ Pathological findings include over proliferation of fibroblasts, excessive deposition of collagen, elastin and proteoglycans in the extracellular matrix.2 Cultured fibroblasts have shown that keloid fibroblasts (KFs) produce up to 12 times more collagen than normal skin fibroblasts in response to TGF-beta. 

Transforming growth factor beta (TGF-β) plays an important role in cell proliferation, apoptosis and differentiation. There is evidence of pre-transcriptional regulation impairments of collagen I production in KFs, with increased synthesis of type I procollagen associated with an increase in its messenger RNA (mRNA) levels.^3^ Surgical excision alone is rarely curative with recurrence rates ranging between 45 and 100%,^4^ frequently associated with stronger collagen buildup and a larger lesion formation to the displeasure of physicians and patients.^5^ Ionizing irradiation as an adjuvant therapy to surgical resection of earlobe keloids, has been historically documented in numerous reports.^6^^,^^7^ Radiotherapy inhibits inflammation, possibly by impairing immune cell function and formation of neovasculature.^8^ Current evidence suggests that the risk of recurrence after pathological scar excision can be reduced after postoperative radiotherapy.^9^

The recurrence rate of ear keloids after surgery and radiation therapy has been estimated in 14.0% (Range: 2.8-33.3%).^10^ The success ratio varies in literature because each study has a different radiation dose, fractionation regimen, field size, or treatment depth. The purpose of this study was to describe our experience treating earlobe keloids with surgical excision followed by high-dose-rate brachytherapy (HDR-BT) and present our outcomes in terms of recurrence, complications and aesthetical result satisfaction.

## MATERIALS AND METHODS

We performed a retrospective chart review and follow-up of 14 earlobe keloids found in 14 patients treated with surgical excision followed by HDR-BT, between January 2015 and May 2016. Information was obtained using institutional electronic medical charts system and paper charts. Data was unidentified, coded and securely saved in a worksheet database in Microsoft® Excel® 2019 software. Database included age, gender, Fitzpatrick skin type, laterality, lesion size and description, comorbidity, operative times, blood loss, and follow-up visits information. Outcomes were assessed in terms of keloid recurrence after completing a full period of follow-up and patient subjective satisfaction level with aesthetic result. 

Complications were defined as surgical wound dehiscence, bleeding, infection and skin necrosis. Postoperative visits were done as clinically indicated during the first 2 weeks, and follow-up visits took place at 3, 6, 12, and 24 months. We performed a descriptive statistical analysis using Microsoft® Excel®, and a stratified data analysis using Graphpad™ Prism® software including patients of any gender, age between 18 and 65 years old, American Society of Anesthesiology (ASA) class I and III, clinical diagnosis of earlobe keloid of 10 mm or more in its wider diameter. A keloid was defined as an area of proliferation of fibrous tissue at the site of a scar or skin injury that grows beyond the original margins of the scar and is possibly associated with erythema, pruritus, pain or paresthesia.

**Fig. 1:**
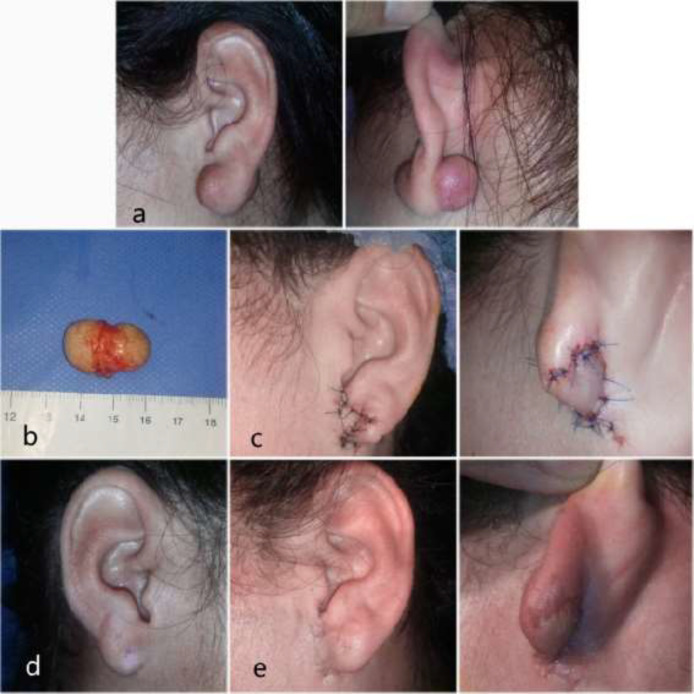
1a. Preoperative views. 1b. Is important to completely remove dumbbell-shaped keloids that literally perforate the earlobe. 1c. Immediate postoperative views. Healthy skin spared from the lateral aspect of the earlobe is used to create a rotational flap to reconstruct the earlobe. 1d. Contralateral earlobe for aesthetical comparison. 1e. Postoperative views 3 months later

**Fig. 2:**
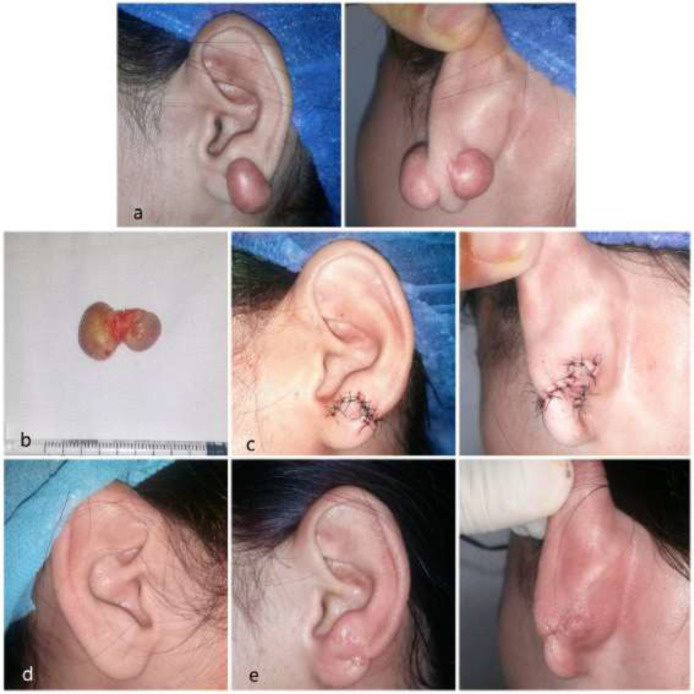
2a. Preoperative views. 2b. Completely resected dumbbell-shaped keloid. 2c. Immediate postoperative views, showing earlobe reconstruction. 2d. Contralateral earlobe for aesthetical comparison. 2e. Postoperative views 1 month later

Patients with ASA IV, keloid location other than earlobe, lesion size less than 10 mm, history of earlobe keloid recurrence, evidence of skin malignancy, history of diabetes mellitus, collagen disease, head or neck radiotherapy, and pregnant women were excluded. Preoperative pictures were taken (Figures 1 and 2). Surgical excision consisted of scalpel “in-bloc” lesion removal, under infiltrative local anesthesia with 2% lidocaine, followed by immediate earlobe reconstruction. Surgical technique was chosen based on the gross appearance of the keloid.^11^

Then, patients were taken to radiotherapy department to start HDR-BT. Protocol consisted of a total dose of 12 Gy, fractionated in 3 doses of 4 Gy given for 3 days, beginning within the first 24 hours after surgical excision (VS200^TM^ Varisource® Iridium-192 source, Brachyvision® v.10.0.39 treatment planning system). Patients were examined postoperatively for 2 weeks as clinically indicated. Sutures were removed after 14 days. Follow-up data was retrieved after reviewing notes from visits at 3, 6, 12, and 24 months. A patient and observer scar assessment scale (POSAS) was used to evaluate aesthetic results at the final follow-up visit.^12^

Overall opinion score of 1 corresponded to normal skin appearance and 10, the worst possible scar. Keloid recurrence was defined as an indurated elevation, where original keloid was situated that could be associated with redness, pruritus, or pain. Patients with keloid recurrence began a protocol of triamcinolone acetonide infiltration in the deep dermis of 0.1 to 0.2 mL at a 5 mg/mL concentration, every 21 days as clinically indicated. This study was performed after approval from Institutional Review Board (IRB) from Hospital Universitario de Caracas, and fulfills all requirements established at Bolivarian Republic of Venezuela Constitution and Declaration of Helsinki regarding Human Subjects Research. Patients’ treatment has already occurred at the time this study was developed. Being a retrospective data study, informed consent was not needed.

## RESULTS

A total of 14 surgical excisions followed by HDR-BT were performed in 14 patients who have been diagnosed with earlobe keloids, between January 2015 and May 2016, and data was used for analysis. Demographics and perioperative data were listed in Table 1. Mean age was 24 years old (range 18-57 years). Eleven patients (78.57%) were females and the remaining three (21.43%) were males. Thirteen patients (93%) were ASA class I and one patient (7%) ASA II. All keloids were secondary to history of earlobe piercing. Average keloid size was 18 mm (range 10-39 mm). All patients were from Hispanic or African-American descent, with Fitzpatrick type III in two patients (14.28%), IV in seven patients (50%), type V in four patients (28.57%), and VI in one patient (7.14%). 

All procedures were completed without complications. Three patients (21.43%) experienced keloid recurrence, two at 6 months (14.28%), and one at 12 months (7.14%). All patients with recurrence responded favorably to triamcinolone infiltration, with one patient experiencing signs of dermal thinning afterwards. No further surgical excisions were needed. Three patients (21.43%) experienced mild signs of self-limited post-radiation dermatitis within the first 4 weeks, including erythema and desquamation. Conservative management with topical emollient was undertaken with satisfactory results. Self-assessment of aesthetical result was considered “very good” in ten patients (71.43%), as listed in Table 1. 

**Table 1:** Patients demographics, skin type, size of keloid, laterality, perioperative results, follow-up data and management

**Patient**	**Gender**	^a^ **ASA**	**Age**	**Size in mm.**	**Fitzpatrick skin type**	**Location**		**Recurrence**				**Complications**	^b^ **POSAS **	**Follow-up **
						**Right**	**Left**	**3 mos.**	**6 mos.**	**12 mos.**	**24 mos.**			
1	F	I	22	12	V		X					-	2	
2	F	I	25	10	IV	X			X			-	4	Thickness, redness and pain at 6 mos. Triamcinolone infiltration x 3 doses.
3	F	I	26	15	IV	X						-	2	
4	M	I	19	25	IV		X					-	2	
5	F	I	18	39	IV		X					-	2	
6	M	I	22	12	V		X					-	2	
7	F	I	29	12	IV		X					-	2	Squamation after 3/weeks of HDR-BT, topic emollient.
8	F	II	57	14	IV		X					-	2	
9	F	I	22	16	V		X		X			-	4	Thickness, redness and pain at 6 mos. Triamcinolone infiltration x 4 doses.
10	F	I	18	15	V	X						-	2	
11	F	I	18	18	IV	X						-	2	
12	F	I	19	25	III		X					-	4	Redness after 2/weeks of HDR-BT, self-limited.
13	F	I	22	32	III	X						-	2	
14	M	I	21	12	VI		X			X		-	5	Squamation and pruritus after 3/weeks of HDR-BT, topic emollient. Thickness, redness and pain at 12 mos. Triamcinolone infiltration x 4 doses.

^a^ASA American Society of Anesthesiologists. ^b^POSAS Patient and Observer Scar Assessment Scale. Only overall opinion is reflected, a score of 1 corresponds to normal skin appearance and 10 the worst possible scar^.^^12^

## DISCUSSION

The justification for using postoperative radiotherapy after pathological scar resection is to control its recurrence.^8^ In our series, surgical excision followed by 12 Gy irradiation delivered in three fractions over three days resulted in 21% of recurrence, 71.43% of “very good” aesthetical outcomes, and 21.43% of mild post-radiation dermatitis. Keloids experiencing recurrence were successfully treated with infiltration of triamcinolone, avoiding further invasive procedures. Shin *et al.* in a systemic review and meta-analysis of 1105 patients treated for ear keloids after surgical excision, adjuvant radiation therapy was associated with an overall recurrence rate of 14% (95 percent CI, 9.6 to 19.9%; *p*<0.001). The most common dose was either 10 Gy or 15 Gy, administered for two or three days after surgery.^9^

Stahl *et al.* reported a recurrence rate of 26% with keloid resection and a perioperative “sandwich” radiotherapy protocol, that consisted in delivering a total dose of 10 to 12.5 Gy in two fractions including a day before and a day after the operation.^13^ A recent report from Ogawa *et al.*, comparing different radiotherapy protocols after surgical excision, showed that 15 Gy administered in three fractions over three days resulted in a 5.7% recurrence rate, 10 Gy in two fractions over two days resulted in 4.6 to 6.9% recurrence rate, and 8 Gy administered in one fraction was associated with a total recurrence rate of 9.3% with no adverse effects.^9^

Familial aggregation, occurrence in identical twins, Mendelian modes of inheritance, expression studies, and the high prevalence of keloids among different ancestries, all provided strong evidence in favor of genetic factors in keloid formation.^1^ Theories have been proposed pointing toward genetic selection mediated by polymorphism of genes responsible for regulation of TGF-beta family, their receptors and signaling molecules.^14^ From an evolution stand point, keloids might have developed as a defense mechanism among certain individuals living in tropical climates to wall off parasitic infections of the skin, resulting in more adverse scarring. Such gene polymorphisms could explain why other individuals are good healers in similar environmental settings.^15^

A report from Jones *et al.* of keloids treated with surgical excision followed by in-office superficial radiation therapy (18 Gy/3fr/3days) showed a 19% recurrence rate, with a 75% of their sample been self-identified as African-American.^16^ Although authors stated that patients from Asian or African-American origin qualify to be managed in the same manner as patients from other racial backgrounds,^17^ we believe that each case should be individualized, especially dealing with patients with high-risk skin phenotypes (Fitzpatrick IV to VI) and positive history of keloids or hypertrophic scarring. 

## CONCLUSION

Surgical excision followed by HDR-BT for earlobe keloids is feasible, secure, and results are favorable and can be offered as a first line combined treatment. Our main drawback was the small number of cases presented and lack of control groups. More research is granted to establish optimal protocols and indications.
